# Peptide specific antibodies to Epstein Barr Virus and Toxoplasma gondii as markers of psychiatric disorders and suicide behaviors

**DOI:** 10.1192/j.eurpsy.2025.58

**Published:** 2025-08-26

**Authors:** R. Yolken, F. B. Dickerson

**Affiliations:** 1 Pediatrics, Johns Hopkins School of Medicine, Baltimore; 2 Psychology, Sheppard Pratt, Towson, United States

## Abstract

**Abstract:**

Immunoassays such as enzyme-linked immunosorbent assays (ELISAs) provide precise measurements of class-specific antibodies to infectious agents. The application of these assays to blood and cerebrospinal fluid from individuals with psychiatric disorders has supported a role for microbial agents in these conditions. However, standard immunoassays are limited by their capacity to measure antibodies to only a single or a small number of infectious agents and epitopes, restricting the ability to identify differential immune responses.

Recent technological advances now allow for comprehensive profiling of immune responses to multiple infectious agents and their specific antigenic epitopes. These methods enable the investigation of differential immune responses as potential modulators of psychiatric disease risk.

We applied two novel serological platforms to analyze immune responses in blood samples from individuals with various psychiatric disorders and suicidal behaviors, as well as healthy controls. The first method assessed antibodies to >4200 peptides derived from >80 infectious agents immobilized onto a solid-phase surface. These peptides were incubated with small blood volumes, and antibody binding was detected via secondary reactions.

Results demonstrated significant differences in immune responses to *Toxoplasma gondii* and Epstein-Barr virus (EBV) in individuals with schizophrenia and other severe psychiatric disorders. Responses to *T. gondii* were primarily directed at dense granule (GRA) proteins, with multiple GRA peptide reactivities detected even in individuals classified as “seronegative” by standard immunoassays. For EBV, differential responses were observed to ZEBRA and other latency-associated proteins. Findings were corroborated using a phage display system, where peptides derived from viral proteins were expressed on the phage surface.

Interestingly, immune reactivity was not uniformly increased or decreased but varied depending on the specific antigen. These findings suggest that psychiatric disorder-associated immune alterations may not merely reflect pathogen exposure but also differences in immune response patterns. Further investigation of these differential responses could inform novel diagnostic, preventive, and therapeutic strategies for severe psychiatric disorders.

**Disclosure of Interest:**

None Declared

